# Exploring Information and Communication Theories for Synthetic Cell Research

**DOI:** 10.3389/fbioe.2022.927156

**Published:** 2022-07-14

**Authors:** Pasquale Stano

**Affiliations:** Department of Biological and Environmental Sciences and Technologies (DiSTeBA), University of Salento, Lecce, Italy

**Keywords:** synthetic cells, artificial cells, bottom-up synthetic biology, information, communication

## 1 Communicating Synthetic Cells

**FIGURE 1:**
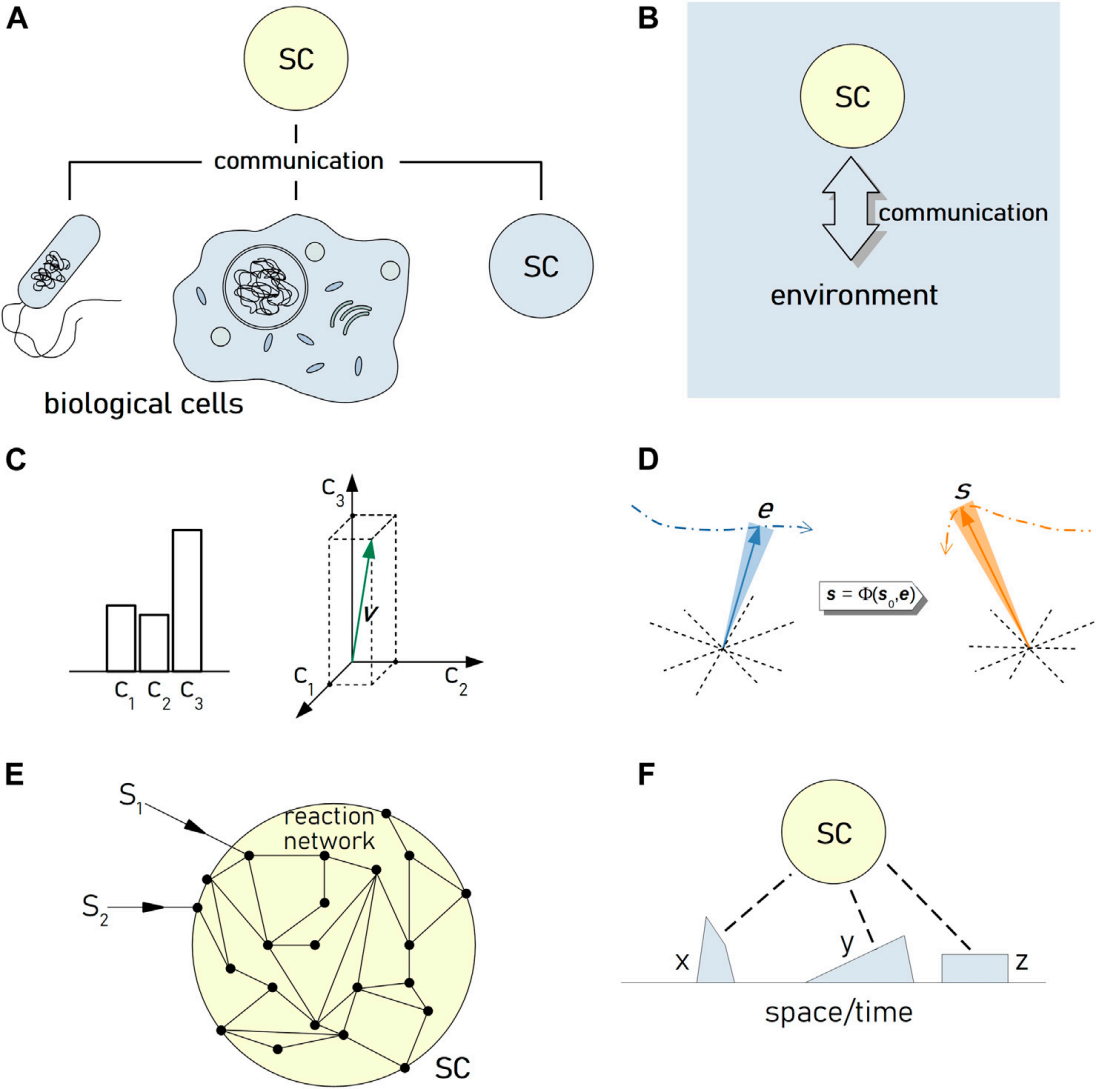
Information and communication in SC research. **(A)** Several reports have demonstrated that SCs can send or receive chemical signals from biological cells or from other SCs. **(B)** Systems based on SCs that receive signals from other cells are equivalent to a generic scenario where a SC (a “system”, an “agent”) exchange signals with its environment. **(C)** Two ways of representing “descriptive” information. On the left, a histogram made of three bars. Bar identity represents the variable that carries information, bar height represents its measure. On the right, the same information is represented as a vector in an informational space; the variables are described by the axes, while the vector components represent the variables’ measures. **(D)** A simplified pictograph representing the trajectories of the environmental vector ***e*** and of the system (the SC) vector ***s*** in their respective multidimensional spaces. The trajectory of ***s*** depends on the initial state of the system (***s***_**0**_) and on the environmental changes (described by ***e***) through the function Φ, which ultimately refers to the “organization” of the system (the set of relations between internal and external variables that determine the system dynamics). **(E)** A SC intended as a reaction network, which can be affected by external stimuli. **(F)** If a SC is *situated* in an environment that can “in-form” it, the resulting dynamics (including the achievement of a predetermined goal) is determined by the environment spatio/temporal pattern.

The now-consolidated interest toward the bottom-up construction of cell-like systems, called “artificial” or “synthetic” cells (SCs), is probably one of the most innovative trends in synthetic biology. SCs are micro-compartmentalized chemical systems built from scratch, which are capable of mimicking cell properties and functions at various degrees of complexity. A quite relevant direction refers to SCs that exchange chemical signals with biological cells or with other SCs present in their surroundings (Figures 1A,B) [Cronin et al. (2006); Gardner et al. (2009); Stano et al. (2012); Lentini et al. (2017); Adamala et al. (2017); Rampioni et al. (2018); Mukwaya et al. (2021); Smith et al. (2022)]. The biotechnological relevance of communicating SCs points, for example, to their potential applications in nanomedicine, as a sort of “smart” programmable agents interfacing biological cells [Chang (1972); Leduc et al. (2007); Krinsky et al. (2018); Ding et al. (2018); Sato et al. (2021)].

The experimental articles reporting advancements in this field constantly increase, as it does the repertoire of available mechanisms for information processing and SC control, suggesting potential near-the-corner applications. We actually assume that impactful studies will be available soon. This opinion paper, however, does not deal with technical advancements. Instead here we will focus on certain theoretical aspects related to how information and communication concepts impinge on SC research, with possible original returns in modeling, interpretations, understandings. These aspects are crucial when the role of SCs (and synthetic biology, in general) is recognized as one approach—the wetware one—to the “Sciences of the Artificial” [Simon (1996); Cordeschi (2002)], the sciences devoted to modelling life and cognition by mean of man-made artifacts. When devoted to these goals, synthetic biology, then, complements well-known fields such as robotics and artificial intelligence, which instead play a role in the hardware and software domains [Damiano et al. (2011); Damiano and Stano (2018, 2021)].

### 1.1 Preludes and Current Descriptions

A preliminary remark is due. Despite several relevant advancements, current built-in-the-lab SCs are not yet alive (in particular, they miss key properties such as autonomy and autopoiesis). Looking at their organization, structure, function, current SCs most resemble machines, although endowed of very peculiar features. This machine-likeness, however, makes it possible to discuss and adapt some established concepts of information and communication theory (ICTs) to SCs by exploiting the rich theoretical framework developed so far, since the age of the first cybernetics^1^. The new tone will be evident, as well as the call to familiarize with new languages^2^.

When experiments about communicating SCs are presented, the discussions generally focus on molecular mechanisms, highlighting the nature of components that successfully concurred to establish a chemical communication, and showing whether the system succeeded or not to perceive signals or to communicate. Although the usual vocabulary of molecular biology is explicitly borrowed from ICTs (signal, receptor, encoding, transcription, translation, etc.), minor relevance is given, to date, to information theoretical aspects. The latter, instead, are the main focus in the recently started field of “molecular communications” (MCs) [Nakano et al. (2013); Nakano (2017)]. Here, communication engineers aim at extending the classical Shannon communication theory to the world of chemical signals, to generate and later exploit a sort of molecular communication protocol. The approach is quite general and can be adapted to any kind of chemical system, including the SCs [Magarini and Stano (2021)].

In this article, instead, we will look to still different descriptions of information and communication, pointing to system dynamics from the viewpoints of heteronomy (or machine-likeness, or “computer Gestalt” perspective—as Varela called it [Varela (1979)], or autonomy (or organism-like). The latter would certainly be more adequate to describe living systems, and it will be shortly commented in Section 4 (as a sort of anticipation to future developments in the field). On the other hand, because current SCs essentially are non-living chemical machines, we maintain that descriptions based on heteronomy are also acceptable and workable for the moment.

The following four topics seem particularly interesting in relation to the above-mentioned goals: 1) the concepts of information and meaning according to Donald M. MacKay [MacKay (1969)]; 2) the cybernetic semiotics discussed by Doede Nauta [Nauta (1972)]; 3) the “in-formation” view of Francisco J. Varela in relation with biological autonomy [Varela (1979)]; and 4) the recent quantitative approach to semantic information proposed by Artemy Kolchinsky and David H. Wolpert [Kolchinsky and Wolpert (2018)].

## 2 Meaning and “Descriptive” Information According to Donald M. MacKay

The information theory developed in the 1950s by Donald M. MacKay (the main representative of the British information tradition [Bar-Hillel (1964); Nauta (1972)]) is strongly linked to cybernetic concepts, and it offers several interesting cues. We will touch here only a couple of them.

The first refers to the technical usage of the word “meaning” when referred to information (semantic aspects of information). The Shannon theory of communication of information explicitly splits the concepts of meaning and information. The theory only refers to the best and faithful way to transfer information (or better, its representation) from a sender to a receiver. The transmitted information is quantified (by the Shannon entropy function) with reference to the difficulty, or improbability, of selecting a specific signal/message from the set of all possible pre-defined signals/messages. What is, instead, the meaning of a signal? MacKay evidences that the meaning is related to what happens in the receiver, in its internal states, once that the signal/message has been received. The meaning should not be confused with the system response^3^, but it refers to how the systems has changed of its propensity to behave in a specific manner. We can add that the system becomes “in-formed” because the signal has caused some changes of internal constraints. MacKay, at this purpose, introduced the concept of conditional “states of readiness”. These are system’s states, defined operatively, to which the system switches according to a matrix which describes the transition probabilities after interacting with signal from the environment. Consequently, MacKay defines the meaning as the selective function that a signal exerts on the set of the states of readiness. The meaning of a signal admits only a relational definition and is context-dependent.

The second cue instead refers to the difference between the “selective” nature of information (when a transmission context is considered) and the quantification of “descriptive” information, the latter being applied to representations of knowledge, with no reference to unexpectedness (which is at the basis of Shannon entropy). MacKay’s descriptive information-content makes use of logons and metrons, which are, respectively, the number of logical classes needed to describe a representation, and the metrical measure of the contribution of each class to the representation. They can be thought as the number of bars and their heights in a histogram representation of a phenomenon, or as the measure of the projections on orthogonal axes of a representational vector in the information space (Figure 1C). It can be noted that when the metron measure is not single-valued, the representation becomes a population of vectors, i.e., it becomes less defined, or fuzzy.

Possible applications of the MacKay theory in SCs research can be considered from the modeling perspective. In contrary to biological cells, SCs modeling does not suffer of lack-of-knowledge about conditions and chemical composition. For example, SCs can be built in order to display state transition dynamics with computable transition probability matrices. Once exposed to a set of signals, meaning can be assigned probabilistically, and then experimentally verified on the basis of predicted vs. observed system trajectories. Moreover, SCs could be ranked based on the descriptive information-amount they are able to perceive. The correlation between the environment information vector and the resulting SC state vector (and their changes) (Figure 1D) can provide a quantitative measure about how SCs cope with changes in their environment, exploiting probabilistic descriptions.

## 3 Cybernetic Semiotics

Based on the combination of Peirce semiotics with systems theory and cybernetics, Doede Nauta “cybernetic semiotics” [Nauta (1972)] is another possible framework for discussing the dynamics of systems (SCs in our case) perceiving their environment. The theory introduces a classification of signs (signal/sign/symbols) and the domains in which they operate (syntactic, semantic, pragmatic). Emphasis is placed on how a system (in the semiotic language, the “interpreter”) that receives information (“information vehicles”) from its environment, copes with it in terms of internal states and operations: the “interpretant” is generated (the effect of information vehicles on the interpreter). The approach somehow resonates with MacKay theories. Nauta also makes a clear distinction between the non-semiotic aspects of transmissional information (à la Shannon) and semiotic processes. The latter refer to the relation between information-vehicles and the cognitive map in the interpreter. Designing SCs that perceive signals or signs of their environment is equivalent to fix what Nauta calls “informational representations”^4^ (e.g., interiorized constraints, patterns, correlations) in the molecular network that constitutes the SC organization (Figure 1E). This analysis makes it clear that SCs whose behavior is not plastic cannot self-generate meanings of the signals. Meanings—in these cases—are imposed by the designer, as it happens with machines. However, thanks to the modularity of synthetic biology molecular sub-systems and devices, meaning can be *de facto* engineered.

## 4 In-Formation

We have seen that the two previous theories ground on the high-level symbolic description of informational processes, often applied when systems are interpreted within a machine paradigm. Maturana and Varela, on the other hand, have presented autopoiesis [Maturana and Varela (1980)] as a theoretical framework for understanding and describing life (and in particular cellular life) in terms of mere interactions between the components of a specific type of network, the one which accomplishes its own production. Because the whole phenomenology of living beings can be reduced to, and explained in terms of, interaction and transformation relations between the network constituents, autopoiesis also provides a self-standing operative description of what life (and cognition) is. No additional “symbolic” explanations are required. In this new perspective, how is communication interpreted? Varela maintains that in contrary to the “computer Gestalt” view, where signals from the environment are considered instructive inputs to the system, the “autonomy” view foresees that the environment and the system are engaged in a coupled dynamics, whereby the system adapts to perturbations (originated in the environment) [Varela (1979)]. The perturbation-adaptation dynamics is constrained by the need of maintaining the autopoietic organization, the perturbations playing a constructive (not instructive) role. Strictly speaking, there are no inputs for an autopoietic network. Autonomous systems deal with their environment in a cognitive (“in-formative”) manner, due to their plasticity. This feature, together with the self-distinction, provides autonomous systems of mind-like character [Varela (1979)]. While the cybernetic interpretation of information and communication remains useful and workable for systems with restricted autonomy, Varela suggested that discussions on information and living systems require a different perspective. This co-dependent, constructive, correlational sense of information {dubbed: “in-formation”, that which is formed within [Bateson (1972)]} should be intended as the structural adaptations of the system to environment perturbations, without the need of symbolic representations and mappings of any sort. The autonomy perspective, whereby a system engages with the environment a co-constructive dynamics can be applied in SC research as a guideline or a framework to for the long-term goal of constructing autopoietic and cognitive, and therefore living, SCs.

## 5 Measuring Semantic Information: An Approach Based on System Dynamics

Kolchinsky and Wolpert (2018) have proposed an operational definition of semantic information based on the coupled dynamics of an environment/system whole [Kolchinsky and Wolpert (2018)]. In particular, they suggested that “semantic information is the syntactic information that a physical system has about its environment which is causally necessary for the system to maintain its own existence”. The approach is quite interesting and may constitutes an original framework in SC modeling. In order to adapt it to current SCs, the condition of “existence”, which recalls the concepts of being alive or at least maintaining a structural or dynamical organization, can be provisionally substituted with less demanding properties, such as performing significant operations (i.e., significant for the observer). Imagine, then, responsive SCs that are situated in an environment where signals have certain spatio-temporal distributions (Figure 1F). The resulting SC behavior, even if determined by the internal chemical network, will depend from the signal patterns too. It is expected that only a sub-set of all possible environmental distributions will best “matches” the constraints *embodied* in the SC organization, leading to successful operations. The amount of semantic information will correspond to the threshold value of environment/SC mutual information, which must be overcome to transition from fail to success^5^.

## 6 Why Should These Topics be of Interest to Synthetic Biologists?

The recent growth of interest in SCs has led to sophisticated systems, constructed from scratch, which are able to exchange chemical signals with the environment and with other cells. One well-known remarkable example is the establishment of bidirectional communication between SCs and living cells [Lentini et al. (2017)], but more recent reports are similarly exciting. These developments mean that SC technology can become a reliable, versatile, powerful and pivotal platform to make one step further, and face either fundamental questions (e.g., what does it mean to be cognitive? Can meaning emerge in artificial systems? Can we engineer it?), either practical applications, such as developing cognitive artificial systems in the chemical domain and interface them with biological systems for whatever purpose—to parallel the impressive developments of artificial intelligence and robotics in the software and hardware domains, aimed at interfacing with humans. To progress the synthetic biology field, we believe that experiments and modeling should be firstly devised within well-thought-out theoretical frameworks, which in simpler cases have been just tacitly understood. For example, it can be agreed that targeting stimulus-response dynamics, even if experimentally challenging, does not require theoretical analyses like the one presented in this article. In contrary, devising an intelligent, or cognitive, or adaptive, or autonomous, or plastic artificial system (just to make some examples) presupposes a preliminary understanding of what these terms mean and under which framework should be understood. Information and communication theories (facing both syntactic and semantic approaches), and other theories as well, can guide more complex implementations, spark novel ideas, be used to confirm or reject working hypotheses.

## 7 Concluding Remarks

A fecund and unexplored area emerges clearly when one considers theoretical implications of SC research in the information and communication arenas, namely the construction of systems that interact with their environment [Damiano and Stano (2018; 2020)]. As mentioned, this research area is already under bright development, but the approaches, the results, and the descriptions have been generally reported and interpreted under the canonical lens of biochemical/molecular biology. This is fine for most of the scopes. In this article we propose that the same research can be also developed into new directions, under the guidance of some theoretical approaches, and try to convince that new concepts, languages, theories can further enrich the synthetic cell research. We hope that this short article will stimulate several scholars to start looking to these opportunities. Indeed, we are convinced that proper developments will definitely contribute not only to reach experimentally valuable goals, but also to advance in the sciences of artificial, with new synthetic approaches to model and understand life and cognition. By means of SC technology, synthetic biology participates to this fundamental challenge with one of the most innovative, multifaceted and versatile instruments.

## Footnotes

1 There is no doubt, in our view, that if SCs had been available at the time, they would have attracted the interest of early cyberneticians. For example, Rosenblueth, Wiener and Bigelow concluded their 1943 seminal article on *Behavior*, *Purpose* and *Teleology* with a visionary remark: “[…] In future years, as the knowledge of colloids and proteins increases, future engineers may attempt the design of robots not only with a behavior, but also with a structure similar to that of a mammal. The ultimate model of a cat is of course another cat, whether it be born of still another cat or synthesized in a laboratory” [Rosenblueth et al. (1943)]. Dealing with cats is premature, yet synthetic biology can partially face the complexity of individual synthetic cells.

2 Quite probably, most experimentally-oriented synthetic biologists have a background in chemistry and/or biology, but soon become interested also in engineering approaches, like those referring to control theory, or in some mathematical/systems biology aspects related to biological system modeling. Expanding the vocabulary to grasp the concepts described in this article is not a different activity than the above-mentioned examples; we believe it will reward interested readers with fascinating insights and inspiration for potential innovative research lines.

3 Simple stimulus-response systems do not have sufficient complexity for a semantic information analysis.

4 They are classified as 1) “implicit” when refer to simple signal/response dynamics; and 2) “concursive”, when the interpreter deploys its cognitive network to orient itself according to received signals or signs.

5 As a brief note connected with Section 3, we mention here a recently published report dealing with a potential “physiosemiotic” interpretation of the Kolchinsky-Wolpert approach [Herrmann-Pillath (2021)].
